# Pivoting from decomposing correlates to developing solutions: An evidence‐based agenda to address drivers of health

**DOI:** 10.1111/1475-6773.13539

**Published:** 2020-08-10

**Authors:** Austin B. Frakt, Ashish K. Jha, Sherry Glied

**Affiliations:** ^1^ VA Boston Healthcare System Boston University School of Public Health Harvard T.H. Chan School of Public Health Boston Massachusetts USA; ^2^ Brown University School of Public Health Providence Rhode Island USA; ^3^ New York University's Robert F. Wagner Graduate School of Public Service New York City New York USA

**Keywords:** delivery of health care, economics, policy, public health, quality of health care

## Abstract

Health is influenced by many factors outside the health system. This is often expressed by decomposing contributors to health into factors that sum to 100 percent. In this commentary, we assess the (few) strengths and (many) limitations of such decompositions. We conclude that they fail to be useful for policy guidance. We conclude by proposing an alternative approach to assessing how various factors affect health: evaluations of interventions.

**What This Study Adds**

1 **What is already known about this topic**

- Health is influenced by many factors outside the health system.
- Several studies formalize this by decomposing contributors to these outcomes into factors that sum to 100 percent.

2 **What this commentary adds**

- Decompositions of health outcomes into contributing factors that add to 100 percent are not as informative and useful as they appear and are highly susceptible to misinterpretation.
- To better inform our decisions about the balance between social policy and health care in improving health, we should focus on interventions.

## 1 INTRODUCTION

Cultural, environmental, political, and economic conditions—the “social determinants”—shape our lives in ways that affect health.^1^, ^2^ A large literature documents associations between social circumstances and health outcomes with a smaller body of work estimating causal effects.^3^ Yet, policy developments in the domains that affect health are largely conducted in isolation, providing little opportunity to allocate limited resources across them to maximize health or well‐being. One reason is that we lack a compelling framework to inform such social policy investments.

Some recent studies suggest we overinvest in health care and underinvest in social needs, which is often interpreted to imply that spending more on social needs would lead to lower health spending.^4^, ^5^ Yet, spending on the two is not necessarily causally related in this way—at current levels of spending in industrialized countries, spending more on social needs may improve health without reducing health care spending.^6^ Thus, maximizing health requires hard choices about how to expend resources.

Despite a dearth of evidence about effects on health and financial return, health care programs and systems are committing resources to social needs. Boston Medical Center, Kaiser Permanente, and the University of Pittsburgh Medical Center are making housing investments^7^; recent legislation permits Medicare Advantage plans to offer supplemental benefits that address social needs^8^; a collaborative project among insurers exchanges best practices in addressing social needs^9^; and some school‐based, educational services are already eligible for Medicaid reimbursement.^10^

There is little doubt that fulfillment of social needs and addressing upstream social determinants are linked to gains in health. The Robert Wood Johnson Foundation‐funded Drivers of Health project considered the evidence on housing and education in particular. Unstable and inadequate housing pose barriers to health.^11^ Improvements in household sanitation and ventilation can reduce the spread of infectious disease^12^; other enhancements can address sources of asthma, lead poisoning, and challenges to mental health.^13^, ^14^ Stable and safe housing is particularly important for the health and safety of children.^15^

Similarly, education is strongly related to health through myriad pathways not all of which are fully understood.^3^ People with at least some college education have mortality rates that are half those of people with at most a high school degree,^16^ and more educated people are healthier by many measures.^17^ Lleras‐Muney^18^ exploited state compulsory education laws enacted between 1915 and 1939 to find that education causally lengthens life spans, though follow‐up studies indicate considerable variation in this effect.^19^, ^20^, ^21^, ^22^, ^23^, ^24^, ^25^, ^26^ Other studies relied on the incentives to pursue more education inherent in a poor labor market^27^ or as a means to avoid the Vietnam War draft,^28^ finding greater education associated with better health and higher likelihood of never smoking.

These plausibly causal linkages between social circumstances and health contrast with evidence that modest increments in health care utilization, unless carefully targeted, have limited effects on health outcomes.^29^ Findings like these are often used to rhetorically buttress the widely held belief that health care plays a small role in the health of the population relative to social determinants.^30^ Several studies formalize this by decomposing contributors to health outcomes into factors that sum to 100 percent (Table 1). In these decompositions, medical care is credited for a 10 percent‐20 percent contribution. Most credit is given to a combination of behaviors or social circumstances.^30^, ^31^, ^32^, ^33^, ^34^, ^35^

**TABLE 1:** Decompositions of contributors to health

Source	Contribution to health by domain				
	Behaviors	Social Circumstances	Environment	Genetics	Medical Care
Booske et al (2010)					
Time span: 2008	30%	40%	10%	—	20%
McGinnis JM, Williams‐Russo P, Knickman JA. (2002)					
Time span: 1980‐2001	40%	15%	5%	30%	10%
Park et al (2015)					
Time span: 2010‐2013	28.9%	45.6%	8.3%	—	17.2%
US Department of Health and Human Services (1980)					
Time span: 1977	48.5%	—	15.8%	26.3%	10.8%

These decompositions are highly influential. McGinnis et al^31^ has been cited over 1100 times and few studies or talks about social determinants of health or social needs fail to reference them. They motivate greater attention to and investment in social needs, which may very well be a good thing. But there are important limitations to these decompositions and the degree to which they can meaningfully inform policy. If we do not understand these limitations, our efforts on addressing social factors are unlikely to maximize health. To this end, the Drivers of Health study, on which we collaborated, included an assessment of the strengths and limitations of prevailing frameworks of determinants of health^34^ and recommendations for new ways to think about them.

## 2 LIMITATIONS OF DECOMPOSITIONS

Decompositions of health outcomes into contributing factors that add to 100 percent are not as informative as they appear and are highly susceptible to misinterpretation. However, they do have reasonable, if circumscribed, applications and interpretations. For example, to aggregate disparate measures of a communities’ or regions’ health‐related factors into overall scores or rankings, one must weigh them. County Health Rankings,^36^ for example, assigns weights of 40 percent, 30 percent, 20 percent, and 10 percent to social and economic factors, health behaviors, clinical care, and the physical environment, respectively. Its creators acknowledge that there is no correct set of weights and whether theirs or any other is “right” depends on how it is used.^37^ They, and we, believe it is fine to use weights such as these for ranking purposes, which is akin to how plan and provider quality measures are aggregated for public reporting and payment.^38^, ^39^ However, in applying weights, users should be explicit that their sizes reflect value judgments about relative importance. Though they may be highly motivating as guidance for areas in which to invest for further research, they are neither indicative of the causal relationships of factors to outcomes nor suitable for evidence‐informed policy making.

Indeed, there are a host of reasons why such decompositions are less meaningful and useful for policy making than widely believed. Here, we emphasize three. First, by construction they omit interactions among factors. Nancy Krieger^40^ showed that bias arises if one fails to include interactions yet forces contributions of factors to sum to 100 percent. See also Remington^41^ and Krieger.^42^ The mathematics and various explanations have been known for decades and can be found in her paper and elsewhere.^43^, ^44^, ^45^ It is analogous to the fact that the probability of A or B is the sum of the probability of A and the probability of B only when A and B are mutually exclusive:

Pr(AorB)=Pr(A)+Pr(B)‐Pr(AandB).

As Weinberg and Zaykin^46^ explain, mortality from most conditions requires multiple causes. For example, developing intellectual disabilities secondary to phenylketonuria “requires both a dysfunctional metabolic gene and an environmental exposure (dietary phenylalanine).” Similarly, many deaths occur because conditions caused by poor health behaviors are not addressed adequately through medical care. These deaths would not occur if both (A) inadequate health care and (B) poor health behaviors did not occur. Mathematically, this is equivalent to Pr(A and B) being nonzero for a sample of deaths. Ignoring this by assuming A and B are mutually exclusive factors and forcing Pr(A) + Pr(B) = 1 introduces bias because it requires estimates of either Pr(A) or Pr(B) or both to absorb the influence of the omitted interaction between A and B.

A second limitation—and one particularly problematic for policy making—is that all decompositions are uninformative about how much is amenable to change. For evidence‐informed policy development (as opposed to research prioritization), one would only consider the health system as playing a role for a health outcome if there is an effective treatment, that is, we have a health intervention that works. Yet, when we blame behaviors, genetics, or the environment for health outcomes to motivate policies in these areas, we typically do so even without known, effective interventions. For example, diet and exercise contribute to obesity and its sequelae. Yet, across myriad interventions, we have had surprisingly little success in changing those behaviors.

Third, decomposition weights are often based on outdated evidence. For example, the work of the DHHS^33^ dates from the mid‐1970s; McGinnis et al^31^ focus on 1980‐2001. The age of evidence is crucial, as many drivers of health change over time. Health care becomes more effective, lifestyles change, and environmental factors evolve, among other things. HIV/AIDS offers one dramatic example. In the 1980s through the mid‐1990s, there was little the health system could do to address it. At that time, AIDS deaths were attributed entirely to behavior, once the vectors of transmission were understood. Today, thanks to effective testing and pharmaceutical prevention, HIV‐positive people can live normal‐length lives. Therefore, an AIDS death now reflects a health system failure, at least to some extent. From hepatitis C to cardiovascular disease to many cancers and other conditions, what were once diseases attributed to lifestyle, genetics, and/or environmental factors are now, in part, due to inadequate health care.

There are other threats to the interpretation, validity, comparability, or utility of decompositions. An unavoidable one, but worth pointing out nonetheless, is that the best evidence available to inform the relationships between social determinants or social needs and health outcomes is often associative. Estimating unbiased, causal effects remains a significant methodological challenge.

Another limitation is some decompositions focus only on mortality,^31^, ^33^ a narrow definition of health. Among those that are broader, there is a heterogeneity of outcomes so that results cannot be easily compared across studies. We cannot view the body of work as replications of the same relationships. Yet another threat to comparability is that some decompositions are based on how much factors contribute to variation in a cross section^33^ and others on associations with changes over time,^47^ two different things. It is possible for a factor to explain little variation in a cross section but have a larger effect size in analyses over time. Attempts to compare these lead to apparent contradictions. For example, in contrast to cross‐sectional decompositions that suggest health care only plays a 10 percent‐20 percent role in our health, other work shows that health care explains 40 percent‐50 percent of longevity gains since the mid‐20th century.^48^

## 3 CONCLUSION

Social factors matter enormously for health, but decomposing health outcomes into drivers that sum to 100 percent is in many ways misleading and unhelpful for policy development. Instead, our view, and that of the Drivers of Health advisory committee co‐chaired by two of us, is that policy should be informed by evidence from interventions. What we need to know is not just how much a factor affects health but how much we can modify it, and by what means, to improve health. That's something only interventions, and good evaluations of them, can tell us.

Unfortunately, we invest very little on studying which policies work and which don't.^49^ Even in health care, less than 0.1 percent of the $3.5 trillion spent is devoted to evaluation.^50^, ^51^ When policy evaluations are conducted, they are rarely done with rigorous designs. Though nearly 80 percent of studies of medical interventions are randomized trials, only 18 percent of studies of US health care policy are,^52^ and many others do not use state‐of‐the‐art quasi‐experimental methods either. When it comes to interventions to address social needs, many studies fail to assess costs (gross and net), which would inform return on investment. However, it can be argued that health‐improving, cost‐effective interventions that address social determinants should be funded even if they don't save money, as we generally do in the case of medical interventions addressing the same problems.^5^, ^8^

It is also important to recognize that interventions can affect a wide range of outcomes, not just health. In evaluating the import of investment in a social program (eg, to expand access to housing), we should measure all the outcomes that improve well‐being. Housing protects people from the elements, provides the stability they need to potentially get a job or build relationships with other people. Even without the health improvements (which are likely there), housing would be enormously beneficial to what people value. Within health care, insurance protects people's financial assets even in cases where it does not improve health.

But, focusing on interventions does not itself provide any guidance as how to prioritize areas in which we could intervene. Should we invest in and study more interventions in housing, education, or health care, for example, or some other area? Here, the Drivers of Health advisory committee could not reach consensus. One conclusion of our work is that there is no single set of priorities that make sense for all people. For some people, especially those with chronic disease, access to health care (eg, through Medicaid expansion) is likely to be a very high priority. For others with different needs, it may be other things, like housing support. So, in developing priories, it is always important to be clear about for whom the priorities apply.

Another challenge in priority development is that there are multiple, valid perspectives on claims to resources. Equity and social justice are as important considerations as value and efficiency. Moreover, these aren't all quantifiable or amenable to ranking. These issues arise even within domains of factors that drive health. For example, a decision to focus on housing would be incomplete. One has to specify exactly how and for whom. There is not a single, best way to do so.

Nevertheless, a focus on the effectiveness of interventions could help address another rhetorical challenge in discussing social needs and determinants. Those conversations often include references to health behaviors or lifestyle, which can easily be interpreted as realms fully within the purview and control of the individual. But the very premise of social determinants is that many apparently autonomous individual choices are, in fact, heavily influenced by social, environmental, and economic circumstances. Individual agency in decisions and changes is less complete than “behaviors” and “lifestyle” imply. This can lead to a temptation, if not a tendency, to blame the victim of social determinants, something we have thankfully learned to do less often for physical diseases that may be no more random, like cancer. Instead, just as in health care, we might make more headway with less distraction through a process of discovery of the means and degree of effective change.

In doing so, however, we should be humble about how much we can improve health. A lot of health isn't explainable or modifiable by any means we know or are likely to discover soon. Forcing known factors to sum to 100 percent provides a false sense of security that we fully know what drives health and where to invest and by how much to do so. A clearer understanding of what we know, and what we don't know, is a better path to ensuring that our investments in social factors truly pay off.

## Supporting information

Author matrix
